# Imbalanced prostanoid release mediates cigarette smoke-induced human pulmonary artery cell proliferation

**DOI:** 10.1186/s12931-022-02056-z

**Published:** 2022-05-28

**Authors:** Abdullah A. Alqarni, Oliver J. Brand, Alice Pasini, Mushabbab Alahmari, Abdulrhman Alghamdi, Linhua Pang

**Affiliations:** 1 Respiratory Medicine Research Group, Academic Unit for Translational Medical Sciences, University of Nottingham School of Medicine, City Hospital Campus, Nottingham, NG5 1PB UK; 2 Department of Respiratory Therapy, Faculty of Medical Rehabilitation Sciences, King Abdulaziz University, Jeddah, Saudi Arabia; 3 Manchester Collaborative Centre for Inflammation Research, The Lydia Becker Institute of Immunology and Inflammation, University of Manchester, Manchester, UK; 4 Department of Electrical, Electronic and Information Engineering “Guglielmo Marconi” (DEI), University of Bologna, Via dell’Università 50, 47522 Cesena, FC Italy; 5 Faculty of Applied Medical Sciences, Department of Respiratory Therapy, University of Bisha, 255, Al Nakhil, Bisha, 67714 Saudi Arabia; 6 Department of Rehabilitation Science, Respiratory Care Program, King Saud University, Riyadh, Saudi Arabia

**Keywords:** Prostanoids, Cigarette smoke, Pulmonary artery cell proliferation, COPD, Pulmonary hypertension

## Abstract

**Background:**

Pulmonary hypertension is a common and serious complication of chronic obstructive pulmonary disease (COPD). Studies suggest that cigarette smoke can initiate pulmonary vascular remodelling by stimulating cell proliferation; however, the underlying cause, particularly the role of vasoactive prostanoids, is unclear. We hypothesize that cigarette smoke extract (CSE) can induce imbalanced vasoactive prostanoid release by differentially modulating the expression of respective synthase genes in human pulmonary artery smooth muscle cells (PASMCs) and endothelial cells (PAECs), thereby contributing to cell proliferation.

**Methods:**

Aqueous CSE was prepared from 3R4F research-grade cigarettes. Human PASMCs and PAECs were treated with or without CSE. Quantitative real-time RT-PCR and Western blotting were used to analyse the mRNA and protein expression of vasoactive prostanoid syhthases. Prostanoid concentration in the medium was measured using ELISA kits. Cell proliferation was assessed using the cell proliferation reagent WST-1.

**Results:**

We demonstrated that CSE induced the expression of cyclooxygenase-2 (COX-2), the rate-limiting enzyme in prostanoid synthesis, in both cell types. In PASMCs, CSE reduced the downstream prostaglandin (PG) I synthase (PGIS) mRNA and protein expression and PGI_2_ production, whereas in PAECs, CSE downregulated PGIS mRNA expression, but PGIS protein was undetectable and CSE had no effect on PGI_2_ production. CSE increased thromboxane (TX) A synthase (TXAS) mRNA expression and TXA_2_ production, despite undetectable TXAS protein in both cell types. CSE also reduced microsomal PGE synthase-1 (mPGES-1) protein expression and PGE_2_ production in PASMCs, but increased PGE_2_ production despite unchanged mPGES-1 protein expression in PAECs. Furthermore, CSE stimulated proliferation of both cell types, which was significantly inhibited by the selective COX-2 inhibitor celecoxib, the PGI_2_ analogue beraprost and the TXA_2_ receptor antagonist daltroban.

**Conclusions:**

These findings provide the first evidence that cigarette smoke can induce imbalanced prostanoid mediator release characterized by the reduced PGI_2_/TXA_2_ ratio and contribute to pulmonary vascular remodelling and suggest that TXA_2_ may represent a novel therapeutic target for pulmonary hypertension in COPD.

## Background

Pulmonary hypertension is a common and serious complication of chronic obstructive pulmonary disease (COPD). It is associated with increased mortality and morbidity [1] and can eventually lead to right ventricular failure and death unless diagnosed early and treated appropriately [2–4]. In recent years, various forms of pulmonary vasoactive agents, including agents targeting prostacyclin pathway (e.g., inhaled prostacyclin analogues and prostacyclin receptor agonist), endothelin receptor antagonists, and nitric oxide pathway enhancers have been approved for the treatment of pulmonary arterial hypertension (group 1 pulmonary hypertension). An oral stimulator of the nitric oxide receptor soluble guanylate cyclase riociguat was recently approved for group 1 and 4 pulmonary hypertension [5–7]. However, these agents are not recommended for pulmonary hypertension due to chronic lung disease and/or hypoxia (group 3) due to lack of studies and no proven benefit in terms of survival [4, 8–11]. It is also thought that pulmonary vasodilator treatment may worsen ventilation/perfusion mismatching in COPD due to the inhibition of hypoxic pulmonary vasoconstriction [12], but there is no direct evidence to support this concept.

Pulmonary hypertension in COPD is characterized by increased pulmonary artery pressure and pulmonary vascular resistance owing to extensive pulmonary vasoconstriction and vascular remodelling, including dysfunction and proliferation of pulmonary artery cells. Studies suggest that cigarette smoking, the major environmental risk factor for COPD, can cause a series of changes in the pulmonary vasculature, including pulmonary artery cell proliferation, which may be critical for pulmonary artery remodelling in COPD- associated pulmonary hypertension [13–15]. Although in vitro studies have shown that cigarette smoke extract (CSE) can induce proliferation of both human and rat pulmonary artery smooth muscle cells (PASMCs) [16, 17], which may eventually lead to vascular remodelling, the effect of CSE on proliferation of human pulmonary artery endothelial cells (PAECs) and the contribution of PAECs and PASMCs, the two key cell types that play a major role in the pathophysiology of pulmonary hypertension, to cigarette smoke-induced pulmonary vascular remodelling in COPD remain unclear.

Prostanoids are a group of vasoactive lipid mediators, including prostaglandins (PGs) and thromboxane (TX), that are synthesized from membrane-derived arachidonic acid (AA). AA is converted to unstable PGH_2_ by the rate-limiting PGH synthase (cyclooxygenase (COX)), which exists in two isoforms, the constitutive COX-1 and the inducible COX-2 (coded by prostaglandin-endoperoxide synthase 2 (*PTGS2*) gene). PGH_2_ is subsequently converted to main biologically functional prostanoids PGI_2_ (prostacyclin), TXA_2_ and PGE_2_ via their respective synthases PGI-synthase (PGIS, coded by *PTGIS* gene), TXA synthase (TXAS, coded by *TBXAS1* gene), PGE synthases (PGES). Among the three different subtypes of PGES (microsomal PGE synthase-1 (mPGES-1), mPGES-2 and cytosolic PGE synthase (cPGES)), mPGES-1 is considered the key enzyme for regulating PGE_2_ [18–20]. PGI_2_ and TXA_2_ have opposing effects (anti-proliferative/vasodilatory *vs* proliferative/vasoconstrictive on pulmonary vasculature). Reduced PGIS expression has been observed in the lung tissue of patients with group 1 pulmonary hypertension. Importantly, this deficiency of PGIS expression is associated with pulmonary vascular remodelling [21], suggesting that reduced PGIS expression and PGI_2_ release may serve as a potential marker for dysfunction of pulmonary artery cells and vascular remodelling in patients with group 1 pulmonary hypertension. Although the role of PGE_2_ in pulmonary hypertension is unclear, previous studies showed that circulating PGE_2_ levels are reduced in patients with group 1 pulmonary hypertension [22] and that the PGE_2_ receptor 2 (EP2) is an important negative modulator of PASMC proliferation [23], suggesting a possible role of PGE_2_ in the development of group 1 pulmonary hypertension. Although an imbalance between the excretion of TXA_2_ and PGI_2_ metabolites [24] and reduction of circulating PGE_2_ levels [22] have previously been reported in patients with group 1 pulmonary hypertension, the imbalance and its functional consequence in the development of pulmonary hypertension in COPD have not been studied.

CSE has been reported to induce COX-2 expression in different cell types including human small airway epithelial cells [25], endothelial cells such as human umbilical vein endothelial cells [26], and human pulmonary microvascular endothelial cells [27]. Although effect of CSE on TXA_2_ and PGE_2_ levels has not been previously investigated in any cell types, including PASMCs and PAECs, reduced PGIS/PGI_2_ has been reported in the lungs of smokers with COPD, as well as in CSE-treated human umbilical vein endothelial cells [28]. The study also showed that umbilical vein endothelial cell stimulation with CSE can induce apoptosis, whilst the use of the PGI_2_ analogue beraprost sodium prevents CSE-induced cell death [28]. The findings of the same group also demonstrated decreased PGI_2_ production and induced apoptosis in the lungs of CSE-treated emphysematous rats [29], suggesting that cigarette smoke can cause cell apoptosis and eventually vascular remodelling possibly by reducing PGIS-derived PGI_2_. However, the effect of CSE on the release of vasoactive prostanoids PGI_2_, TXA_2_, and PGE_2_ and the expression of their respective synthases PGIS, TXAS, and mPGES-1 in PAECs, particularly in vascular smooth muscle cells including PASMCs, is unknown. In addition, the involvement of these vasoactive prostanoid meditators in CSE-induced pulmonary artery cell proliferation has not been previously investigated.

In this study we explored the effect of CSE on vasoactive prostanoid synthase gene expression and mediator release and the role of imbalanced prostanoid mediator release (by the use of agents that target the imbalance prostanoid pathway in CSE-induced human PASMC and PAEC proliferation). We report for the first time that CSE induces imbalanced vasoactive prostanoid mediator release characterized by the reduced PGI_2_/TXA_2_ ratio, likely as a result of an imbalanced expression of PGIS and TXAS in both PASMCs and PAECs, and that the imbalance plays a key role in mediating CSE-induced cell proliferation in both cell types and may thereby contribute to vascular remodelling. Our results strongly suggest that the imbalanced prostanoid release, TXA_2_ in particular, has the potential to be a novel therapeutic target in COPD-associated pulmonary hypertension. We have previously reported some of the results of this study in the form of abstracts [30–32].

## Materials and methods

### Cigarette smoke extract (CSE) preparation

Aqueous CSE from 3R4F research-grade cigarettes (Kentucky Tobacco Research and Development Center, University of Kentucky, USA) was prepared under standardised conditions by bubbling the smoke from two cigarettes at 1 cigarette/min rate into 20 ml low-serum (0.5% foetal bovine serum (FBS)) Dulbecco’s Modified Eagle Medium (DMEM) for PASMCs or endothelial cell growth medium (ECGM) for PAECs. The smoke was drawn through the medium using a vacuum pump (Charles Austen Pumps, UK) with a constant pressure of 0.2 bars to achieve a static burning rate. The resulting CSE was filtered through a 0.22 μm pore size filter and the absorbance at 320 nm was used to measure CSE strength (1.5 = 100%) [33] prior to preparation of the desired concentrations.

### Cell culture

Human PASMCs from three different healthy donors were obtained from Thermo Fisher Scientific (Waltham, MA, USA), Cell Applications (San Diego, CA, USA), and Lonza Group (Basel, Switzerland) and human PAECs, isolated from the main pulmonary artery of a healthy donor, were purchased from PromoCell (Heidelberg, Germany). PASMCs at passage 6 were cultured in growth DMEM (supplemented with 20% FBS) as previously described [34] until 90% confluence, treated with CSE in growth DMEM for 48 h and then with CSE in low serum DMEM (0.5% FBS) for 24 h. PAECs at passage 8 were cultured in ECGM (containing 2% FBS, 0.4% endothelial cell growth supplement, 0.1 ng/ml epidermal growth factor, 1 ng/ml basic fibroblast growth factor, 90 ng/ml heparin and 1 ng/ml hydrocortisone) until 90% confluence and then treated with CSE for 24 h. For cell proliferation study, cells were pre-treated with or without celecoxib, beraprost sodium and daltroban (all from Merck Life Science UK, Dorset, UK) for 1 h before being treated with CSE in growth DMEM and ECGM, respectively, for 24 h. The concentrations of CSE and drugs and treatment time used in this study were determined based on the cytotoxicity assesed by 3-(4,5-dimethylthiazol-2-yl)-2,5- diphenyltetrazolium bromide (MTT) assay (Merck Life Science UK).

### Quantitative real-time RT-PCR (qRT-PCR) analysis

Confluent human PASMCs and PAECs were treated with CSE for 72 h and 24 h, respectively. Total RNA was isolated from the cells using the NucleoSpin® RNA kit following the instructions of the manufacturer (Macherey Nagel, Germany). After total RNA quantification using NanoDrop spectrophotometer (Thermo Fisher Scientific), 1 μg total RNA of each sample was reverse transcribed to cDNA with the high-capacity cDNA reverse transcription kit (Applied Biosystems, USA) [35]. Reversed transcribed cDNA was diluted (1:10 ratio) with nuclease free water. 5 μl of the cDNA sample were amplified using 1 μM primers and the KAPA SYBR® FAST qPCR Kit (Kapa Biosystems, USA). The relative quantification was calculated with the 2^−ΔΔCT^ method using the internal control β_2_-microglobulin (*β2M*) as a reference gene. Data were expressed as fold change over untreated (0% CSE) cells. The primer sequences used are: *PTGS2* (FW): 5'-AAGCAGGCTAATACTGATAGG-3' (RV): 5'-TGTTGAAAAGTAGTTCTGGG-3'; *PTGIS* (FW): 5'-GAAAGACTTTTACAAGGATGGG-3' (RV): 5'-ATTGTTTGATGCTGTTGACC-3'; *TBXAS1* (FW): 5'-CTACTGCAATTACACCACAG-3' (RV): 5'-AAGAGTAAAACCAGGATAGGTC-3'; *β2M* (FW): 5'-AAGGACTGGTCTTTCTATCTC-3' (RV): 5'-GATCCCACTTAACTATCTTGG-3'.

### Western blotting analysis

At the end of the experiment, the cell culture medium was collected for mediator release analysis, and human PASMCs and PAECs were lysed with RIPA buffer. After being collected and quantified for protein concentrations using a bicinchoninic acid (BCA) assay kit (Thermo Fisher Scientific), protein samples were diluted in 4 × Laemmli buffer and boiled for 10 min. 20 μg of total protein were separated with SDS-PAGE and transferred into polyvinylidene fluoride (PVDF) membrane (BioRad, USA). Next, the membrane was blocked with 5% non-fat dry milk (Santa Cruz, USA) in TBS-T (Tris-Buffered Saline with 0.1% Tween 20). The membrane was then probed with specific antibodies recognizing human forms of COX-2 (Cayman Chemical, USA), PGIS (R&D Systems, UK), TXAS (Novus Biologicals, USA), mPGES-1(Cambridge bioscience, UK), and GAPDH (Santa Cruz Biotechnology, USA). ImageJ software (National Institutes of Health, USA) was used to quantify protein band density from Western blot. The values were first normalised to the corresponding GAPDH and then to the control sample as described before [35]. Data were presented as fold changes over control.

### Prostanoid analysis

PGE_2_, 6-keto-PGF1_α_ (stable metabolite of PGI_2_) and TXB_2_ (stable metabolite of TXA_2_) released in the culture medium were quantified using respective ELISA kits (Cayman Chemical), following the manufacturer’s instructions. Data were normalized with total protein and expressed as pg/mg protein.

### Cell proliferation analysis

Cell proliferation of human PASMCs (in growth DMEM) and PAECs (in ECGM) in response to CSE treatment for up to 72 h and 24 h, respectively, was assessed using the cell proliferation reagent WST-1 (water solubale tetrazolium salt 1) following the instructions of the manufacturer (Sigma-Aldrich) as described before [36]. Data were expressed as relative proliferation (% change over control).

### Statistical analysis

Data were expressed as mean ± SEM. GraphPad Prism 8 was used for statistical analysis. An unpaired Student's t test was carried out to compare two data sets. ANOVA followed by a t-test was performed to compare the differences between control and specific treated groups. *P* < 0.05 was regarded as statistically significant.

## Results

### CSE differentially regulates the mRNA and protein expression of *PTGS2*, *PTGIS*, and *TBXAS1*

**Fig. 1:**
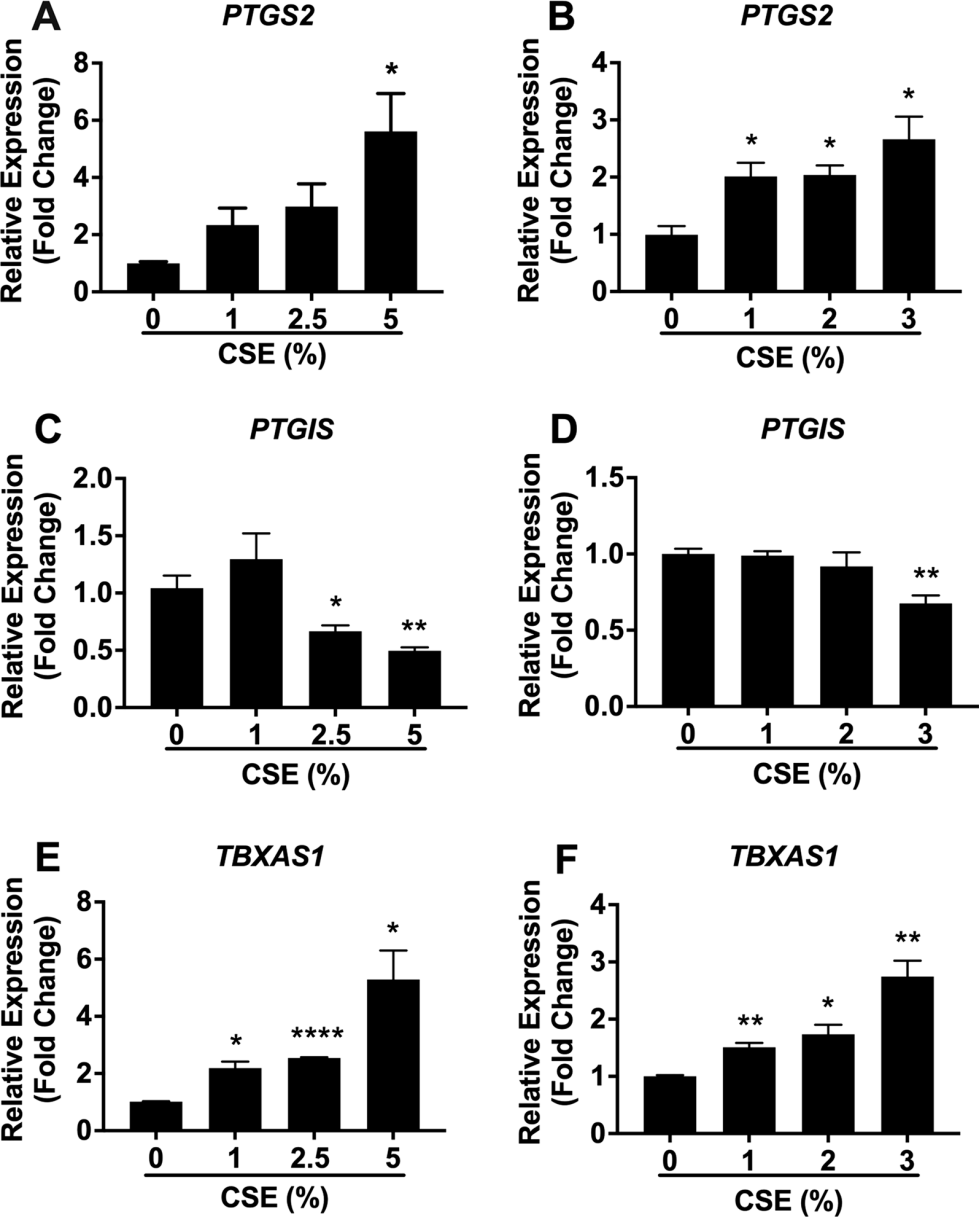
CSE treatment differentially modulates the mRNA expression of *PTGS2*, *PTGIS*, and *TBXAS1* in human PASMCs and PAECs. Confluent human PASMCs (**A**, **C**, and **E**) and PAECs (**B**, **D**, and **F**) were treated with different concentrations of CSE for 72 h and 24 h, respectively. Total RNA was isolated, and mRNA levels of *PTGS2* (**A**, **B**), *PTGIS* (**C**, **D**), *TBXAS1* (**E**, **F**), and the internal control *β2M* were determined by real-time RT- PCR. Results are calculated as the ratio of target gene mRNA and *β2M* mRNA and are expressed as fold change over untreated (0% CSE) cells. Each data point represents mean ± SEM from three independent experiments using PASMCs (**A**, **C**, and **E)** from three different donors and PAECs (**B**, **D**, and **F**) from one donor. *P < 0.05, **P < 0.01, ****P < 0.0001 compared with corresponding untreated cells

COX-2, PGIS and TXAS are vasoactive enzymes responsible for the production of their downstream prostanoid products that have potent anti-proliferative (e.g. PGE_2_ and PGI_2_) and proliferative (e.g. TXA_2_) properties in pulmonary vasculature. However, the effect of CSE on the mRNA expression of their respective genes in both human PASMCs and PAECs is largely unknown. As shown in Fig. 1A, B, *PTGS2* mRNA was basally expressed under unstimulated condition in both human PASMCs and PAECs. In PASMCs (Fig. 1A), treatment with 1% and 2.5% CSE for 72 h had no effect, but treatment with 5% CSE significantly upregulated *PTGS2* mRNA expression (fold change 5.6 ± 1.3) compared with untreated cells. In PAECs (Fig. 1B), treatment with 1, 2, and 3% CSE for 24 h all significantly upregulated *PTGS2* mRNA expression (fold change 2.0 ± 0.28, 2.0 ± 0.17, and 2.7 ± 0.40, respectively, p < 0.01 for all). *PTGIS* and *TBXAS1* mRNA was also detectable in both cell types under unstimulated condition (Fig. 1C–F). In PASMCs (Fig. 1C), treatment with 2.5% and 5% (but not 1%) CSE significantly reduced *PTGIS* mRNA expression compared with untreated cells (fold change 0.7 ± 0.05, p < 0.05 and fold change 0.5 ± 0.03, p < 0.01, respectively). Similarly, stimulation with 3% CSE caused a significant reduction of *PTGIS* mRNA expression (fold changed 0.7 ± 0.05, p < 0.01) in PAECs, whereas 1% and 2% CSE had no effect (Fig. 1D). Similar concentration-dependent increase of *TBXAS1* mRNA expression by CSE treatment was also observed in both PASMCs (Fig. 1E) and PAECs (Fig. 1F). The mRNA data suggest that CSE induces an imbalanced expression of the *PTGIS* gene (downregulated) and the *TBXAS1* gene (upregulated) in both cell types.

**Fig. 2:**
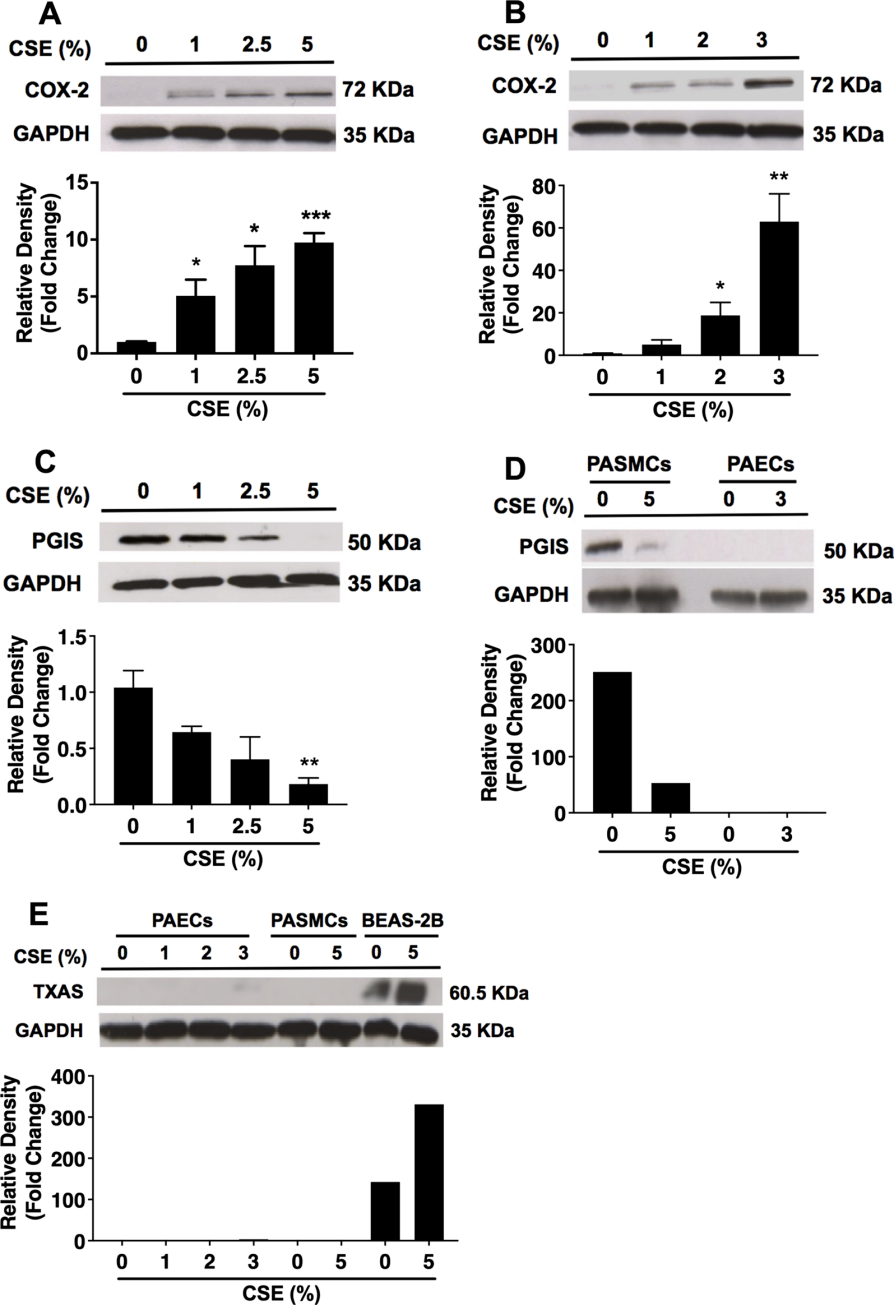
CSE treatment differentially modulates the protein expression of key enzymes of prostanoid synthesis in human PASMCs and PAECs. Confluent human PASMCs (**A**, **C**, and **E**) and PAECs (**B**,** D**, and **E**) were treated with different concentrations of CSE for 72 h and 24 h, respectively. Total cell lysates were collected, and protein levels of COX-2 (**A**, **B**), PGIS (**C**, **D**), TXAS (**E**), and the internal control GAPDH were analyzed by Western blot. Total cell lysates from immortalized human bronchial epithelial (BEAS-2B) cells treated with or without CSE were used as a positive control (**E**). Irrelavant parts of the Western blotting images were cropped. Optical densitometry analysis of Western blotting bands was then conducted. Results are calculated as the ratio of target protein and GAPDH and are expressed as fold change over untreated (0% CSE) cells. Each data point represents mean ± SEM from three independent experiments using PASMCs (**A**,** C**, and **E**) from three different donors and PAECs (**B**, **D**, and **E**) from one donor. *P < 0.05, **P < 0.01, ***P < 0.001 compared with corresponding untreated cells

We next questioned whether the differentially regulated mRNA expression of *PTGS2*, *PTGIS*, and *TBXAS1* by CSE could be translated into similar changes of protein expression of the respective genes. Although *PTGS2* mRNA was detectable, COX-2 protein was not detected by Western blotting in unstimulated PASMCs and PAECs (Fig. 2A, B). Consistent with upregulated *PTGS2* mRNA expression, CSE treatment also induced a marked upregulation of COX-2 protein in a concentration-dependent manner in both cell types (Fig. 2A, B). In contrast to COX-2, PGIS protein was abundantly expressed in unstimulated PASMCs, but the expression was reduced in a concentration-dependent manner (significant at 5%) by CSE treatment in PASMCs (Fig. 2C). Unexpectedly, PGIS protein was not detected under both unstimulated and CSE-stimulated conditions in PAECs (Fig. 2D); and TXAS protein was also not detected with or without CSE treatment in both PASMCs and PAECs (Fig. 2E). The protein data suggest that CSE may induce an imbalanced expression of PGIS and TXAS at least in PASMCs by reducing PGIS expression.

### CSE induces opposing effects on PGE_2_ production and an imbalanced PGI_2_/TXA_2_ release

**Fig. 3:**
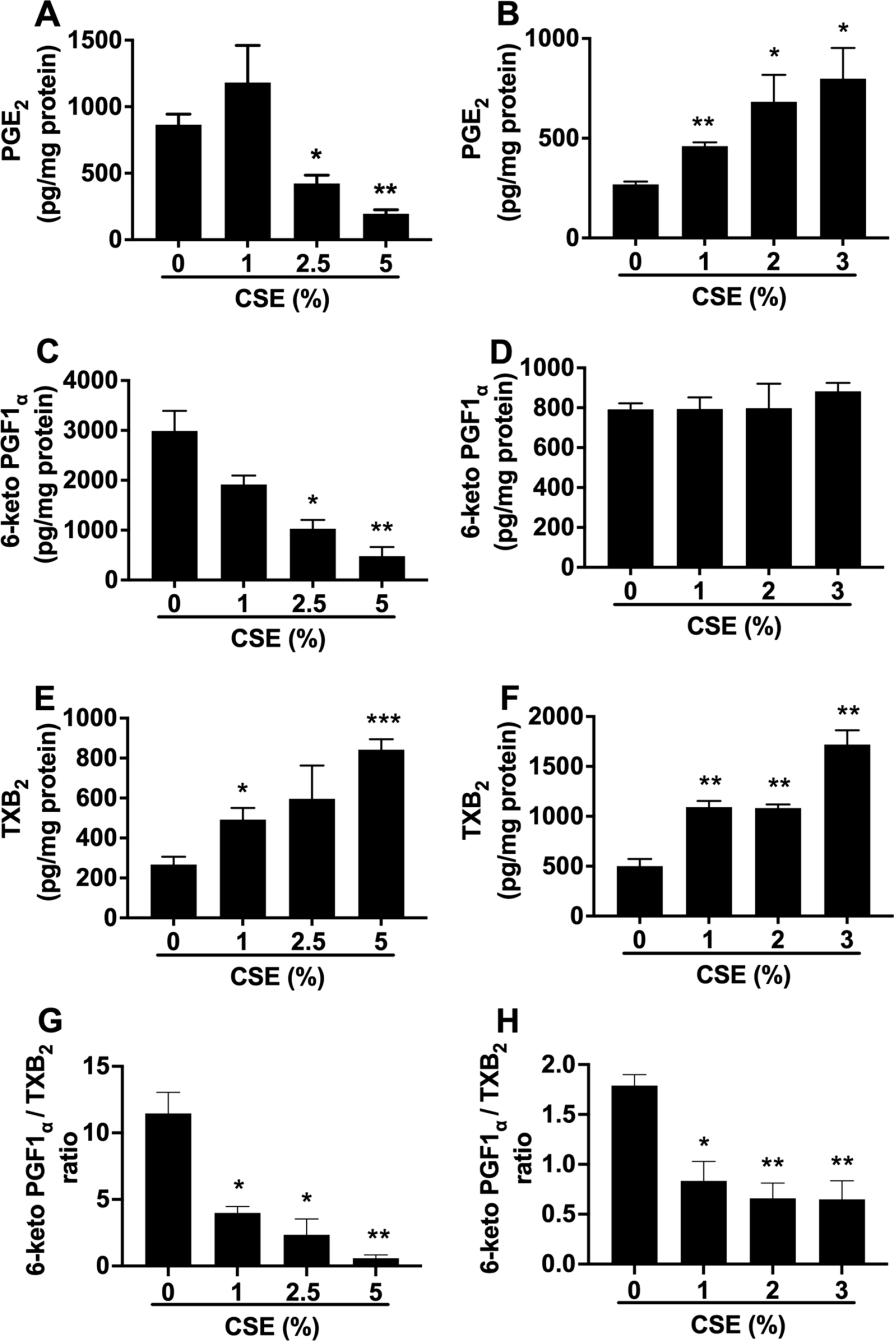
CSE treatment induces 6-keto PGF1_α_/TXB_2_ imbalance and elicits opposing effects on PGE_2_ production in human PASMCs and PAECs. Confluent human PASMCs (**A, C, E,** and **G**) and PAECs (**B, D, F,** and **H**) were treated with different concentrations of CSE for 72 h and 24 h, respectively. Medium was collected, and levels of PGE_2_ (**A**, **B**), 6-keto PGF1_α_ (**C**, **D**) and TXB_2_ (**E**, **F**) were determined by ELISA and 6-keto PGF1_α_/TXB_2_ ratio (**G** and **H**) was calculated. Results were normalised with total cell protein and are expressed as pg/mg protein (**A**–**F**) or 6-keto PGF1_α_/TXB_2_ ratio (**G**, **H**). Each data point represents mean ± SEM from three independent experiments using PASMCs (**A**, **C**, **E** and **G**) from three different donors and PAECs (**B**, **D**, **F**, and **H**) from one donor. *P < 0.05, **P < 0.01, ***P < 0.001 compared with corresponding untreated cells

We next went on to explore whether the altered mRNA and protein expression of the vasoactive enzymes could lead to imbalanced prostanoid mediator release. COX-2 is the rate-limiting enzyme in the synthesis of its downstream prostanoid products, such as PGE_2_, PGI_2_, and TXA_2_. PGE_2_, a major prostanoid product, is commonly used as an indication for COX-2 activity. As shown in Fig. 3A, PGE_2_ was produced in unstimulated PASMCs (863.2 ± 79.6 pg/mg protein). Treatment with 1% CSE had no effect on PGE_2_ production; but surprisingly, treatment with 2.5% and 5% CSE resulted in a significant reduction in PGE_2_ production (422.7 ± 63.4 pg/mg protein and 195.1 ± 30.0 pg/mg protein, respectively) compared with unstimulated cells. PGE_2_ was also produced at basal levels in human PAECs (263.5 ± 32.0 pg/mg protein) (Fig. 3B). In contrast to the findings from PASMCs, CSE treatment at all concentrations significantly increased PGE_2_ production in a concentration-dependent manner (Fig. 3B).

Both PGI_2_ (measured as the stable metabolite 6-keto PGF1_α_) and TXA_2_ (measured as the stable metabolite TXB_2_) were produced in control PASMCs and PAECs (Fig. 3C–F) and the ratio of PGI_2_/TXA_2_ (calculated as 6-keto PGF1_α_/TXB_2_) was around 12 and 1.75 for PASMCs and PAECs (Fig. 3G, H), respectively. Treatment of PASMCs with CSE significantly and concentration-dependently reduced PGI_2_ production, but increased TXA_2_ production (Fig. 3C and E). As a result, PGI_2_/TXA_2_ ratio was markedly reduced after treatment of cells with 1%, 2.5% and 5% CSE (4.0 ± 0.5, p < 0.05, 2.3 ± 1.2, p < 0.05 and 0.6 ± 0.2, p < 0.01, respectively) (Fig. 3G). Interestingly, treatment of PAECs with CSE had no effect on PGI_2_ production, but significantly increased TXA_2_ production in a concentration-dependent manner (Fig. 3D and F), thereby resulting in a significantly reduced PGI_2_/TXA_2_ ratio by 1%, 2% and 3% CSE (0.8 ± 0.2, p < 0.05, 0.6 ± 0.1 and 0.6 ± 0.1, p < 0.01, respectively) (Fig. 3H).

**Fig. 4:**
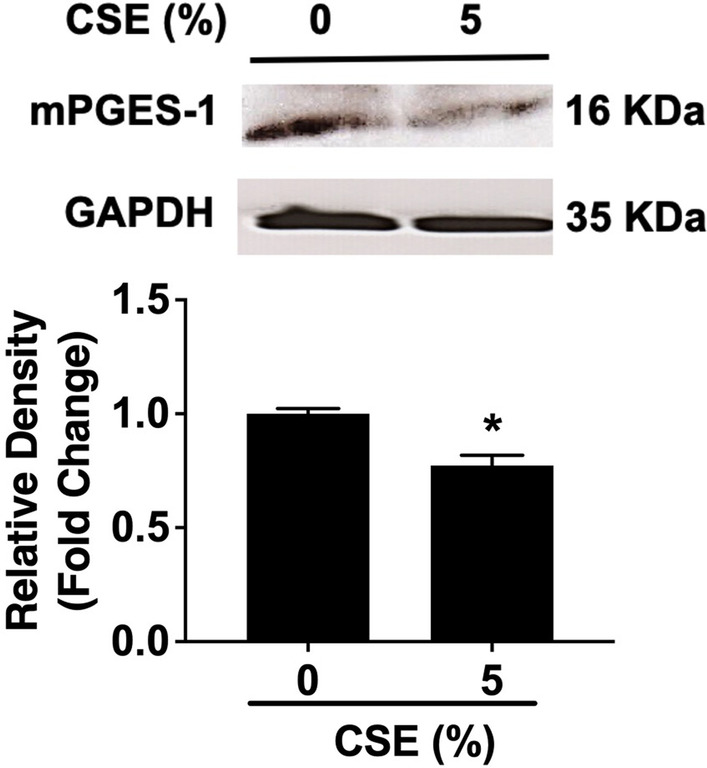
CSE treatment downregulates the protein expression of mPGES-1 in human PASMCs. Confluent human PASMCs were treated with CSE (5%) for 72 h. Total cell lysates were collected, and protein levels of mPGES-1 and the internal control GAPDH were analyzed by Western blot. Irrelavant parts of the Western blotting images were cropped. Optical densitometry analysis of Western blotting bands was then conducted. Results are calculated as the ratio of mPGES-1 and GAPDH and are expressed as fold change over untreated (0% CSE) cells. Each data point represents mean ± SEM from three independent experiments using PASMCs from three different donors. *P < 0.05 compared with untreated cells

Although CSE induced COX-2 expression in both PASMCs and PAECs (Fig. 2A, B) and increased PGE_2_ production in PAECs (Fig. 3B), PGE_2_ production was unexpectedly reduced by CSE in PASMC (Fig. 3A), suggesting that CSE may regulate the expression of mPGES-1, the enzyme responsible for PGE_2_ production downstream of COX-2, differently in the two cell types. Therefore, the effect of CSE on mPGES-1 protein expression was analyzed. It was revealed that mPGES-1 was basally expressed in PASMCs and the expression was significantly reduced by treatment with 5% CSE (Fig. 4). In contrast, mPGES-1 was also basally expressed in PAECs and no change of the expression was observed after treatment with CSE (data not shown).

These data altogether suggest that CSE induces an imbalanced prostanoid release characterized by the reduced PGI_2_/TXA_2_ ratio, likely as a result of an imbalanced expression of PGIS and TXAS in PASMCs and PAECs, and that this imbalance may promote proliferation of both cell types, thereby contributing to pulmonary vascular remodelling in COPD.

### CSE treatment stimulates proliferation of PASMCs and PAECs, and the proliferation is inhibited by celecoxib, beraprost sodium and daltroban

**Fig. 5:**
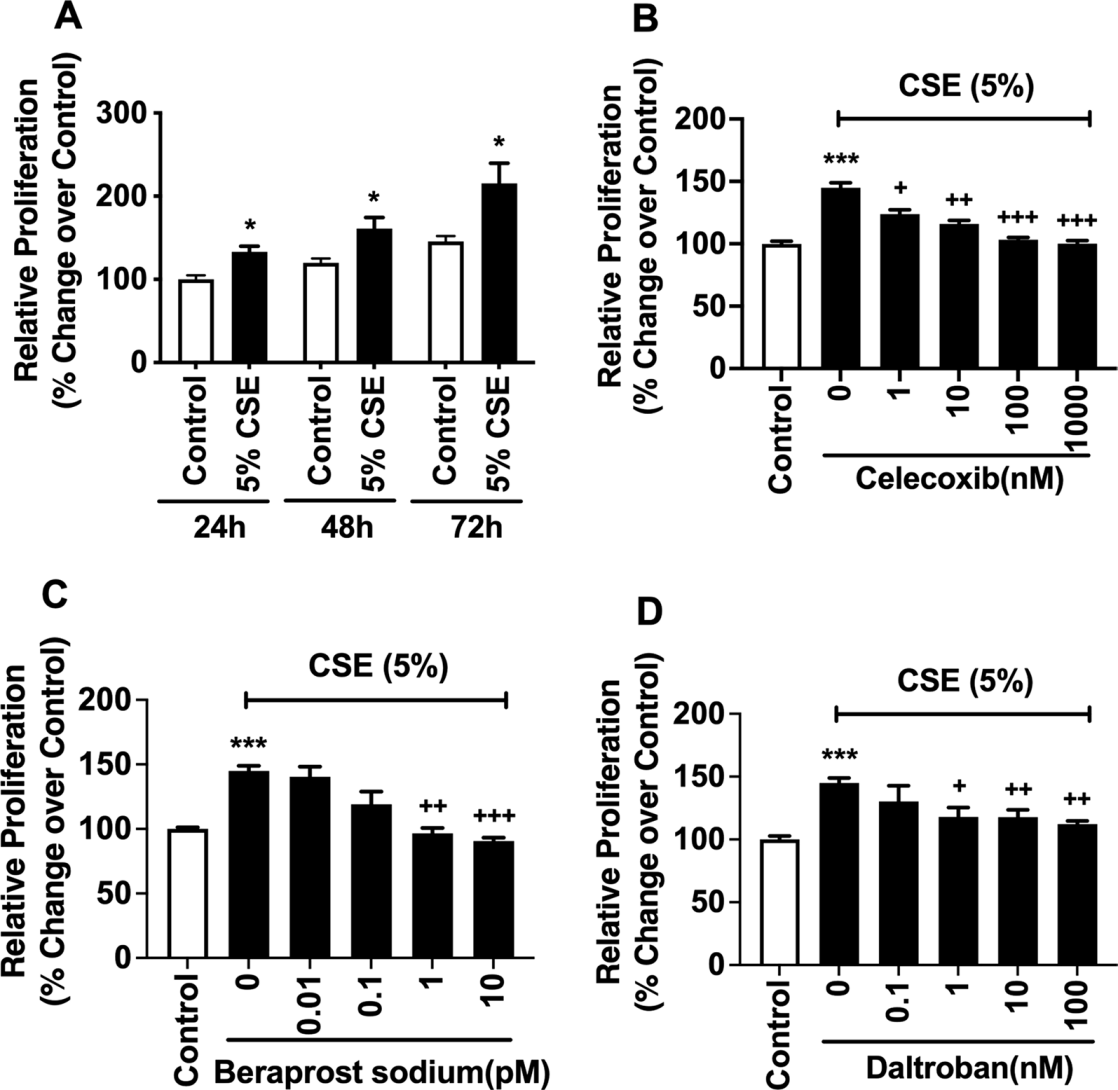
CSE treatment stimulates proliferation of human PASMCs, and celecoxib, beraprost sodium and daltroban inhibit the proliferation. (**A**) confluent human PASMCs were treated with or without CSE for up to 72 h, and then WST-1 assay was conducted. (**B**–**D**) confluent human PASMCs were pre-treated with or without different concentrations of celecoxib (**B**), beraprost sodium (**C**) and daltroban (**D)** for 1 h before being treated with CSE for 24 h. WST-1 was then conducted. Data are expressed as relative proliferation (% change over control). Each data point represents mean ± SEM from three independent experiments using PASMCs from three different donors. *P < 0.05, ***P < 0.001 compared with control; ^+^*P* < 0.05, ^++^*P* < 0.01, ^+++^*P* < 0.001 compared with CSE alone

**Fig. 6:**
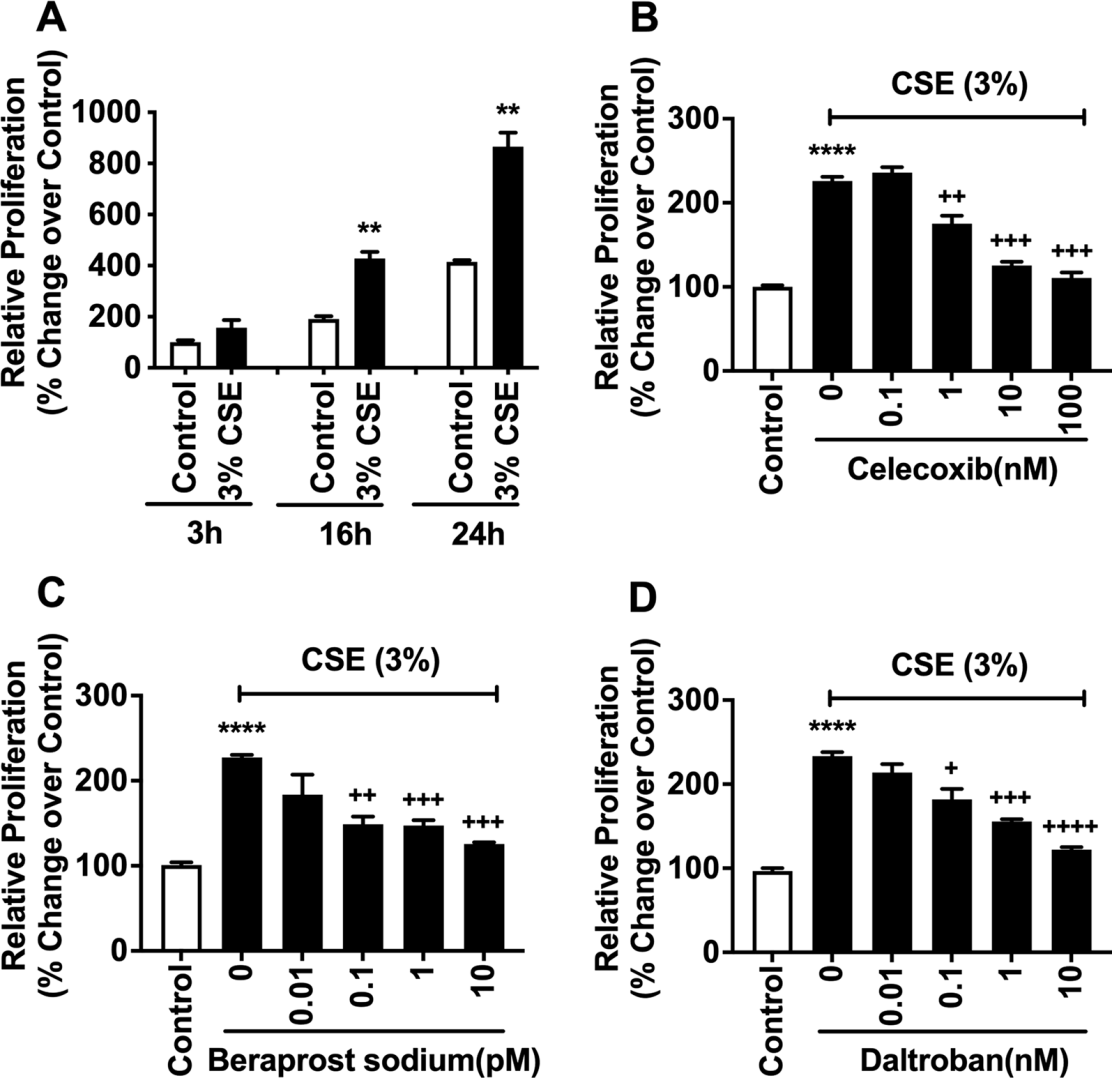
CSE treatment stimulates proliferation of human PAECs, and celecoxib, beraprost sodium, and daltroban potently inhibit the proliferation. (**A**) confluent human PAECs were treated with or without CSE for up to 24 h, and then WST-1 assay was conducted. (**B**–**D**) confluent human PAECs were pre-treated with or without different concentrations of celecoxib (**B**), beraprost sodium (**C**), and daltroban (**D**) for 1 h before being treated with CSE for 24 h. WST-1 was then conducted. Data are expressed as relative proliferation (% change over control). Each data point represents mean ± SEM from three independent experiments using PAECs from one donor **P < 0.01, ****P < 0.0001 compared with control; ^+^*P* < 0.05, ^++^*P* < 0.01, ^+++^*P* < 0.001, ^++++^*P* < 0.0001 compared with CSE alone

We then assessed whether CSE treatment stimulated proliferation of PASMCs. It was found that CSE at 5% significantly stimulated proliferation of PASMCs at all time points tested compared with the corresponding control (Fig. 5A). To test whether CSE-induced COX-2 expression plays a role in PASMC proliferation, the effect of celecoxib, a selective COX-2 inhibitor, on CSE-induced cell proliferation was assessed. The results showed that CSE treatment significantly increased cell proliferation compared with control, and this effect was inhibited by celecoxib in a concentration-dependent manner (Fig. 5B). Since mPGES-1 expression and PGE_2_ release were reduced by CSE, the results suggest the release of prostanoids other than PGE_2_ downstream of COX-2 plays a role in CSE-induced PASMC proliferation. As CSE reduced the release of the anti-proliferative prostanoid PGI_2_ and increased the release of the proliferative TXA_2_ downstream of COX-2, the effect of the stable PGI_2_ analogue beraprost sodium and the selective TXA_2_ receptor antagonist daltroban on CSE-induced PASMC proliferation was investigated. As shown in Fig. 5C, D, both beraprost sodium and daltroban significantly inhibited CSE-induced PASMC proliferation in a concentration-dependent manner, suggesting reduced PGI_2_ and increased TXA_2_ both contribute to CSE-induced PASMC proliferation. Similarly, CSE at 3% also significantly increased PAEC proliferation at 16 and 24 h compared with the corresponding control (Fig. 6A), and the increase at 24 h was significantly and concentration-dependently inhibited by celecoxib (Fig. 6B), beraprost sodium (Fig. 6C) and daltroban (Fig. 6D), but not by the mPGES-1 inhibitor PF-03549184 (data not shown). The results suggest that reduced PGI_2_ and increased TXA_2_, but not increased PGE_2_, contribute to CSE-induced PAEC proliferation.

**Fig. 7:**
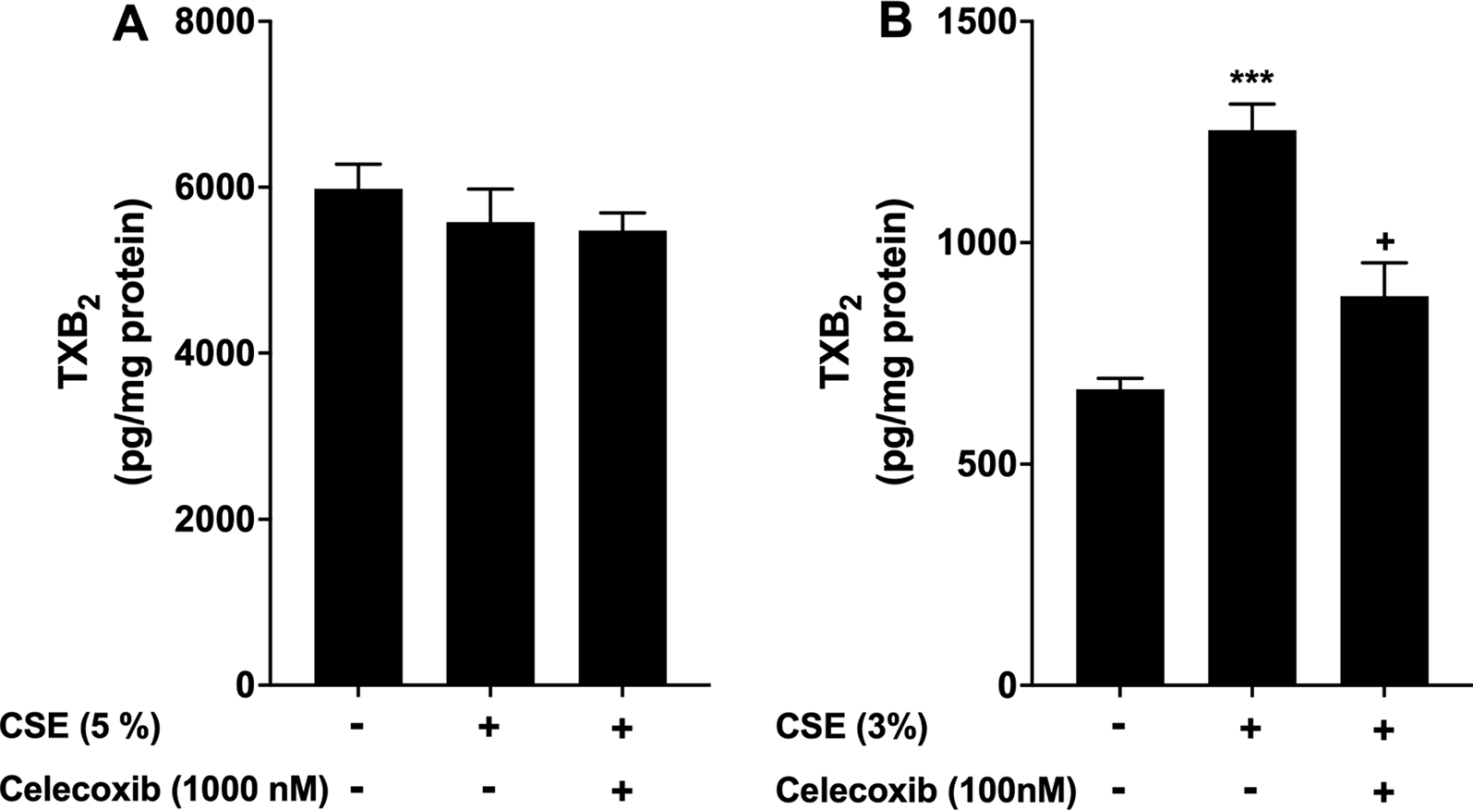
Selective COX-2 inhibition reduces CSE-induced TXB_2_ production in human PAECs, but not in PASMCs. Confluent human PASMCs (**A)** and PAECs (**B)** were pre-treated with one concentration of celecoxib for 1 h before being treated with CSE for 24 h. Medium was collected, and TXB_2_ concentration was determined by ELISA. Results were normalised with total cell protein and are expressed as pg/mg protein. Each data point represents mean ± SEM from three independent experiments using PASMCs (**A)** from three different donors and PAECs (**B)** from one donor. ***P < 0.001 compared with corresponding untreated cells; ^+^*P* < 0.05 compared with CSE alone

The COX-2 inhibitor celecoxib could inhibit the release of all prostanoids downstream of CSE-induced COX-2, including PGE_2,_ PGI_2_ and TXA_2_. But in PASMCs, the release of PGE_2,_and PGI_2_ was reduced by CSE, and in PAECs, PGI_2_ release was not affected by CSE and the mPGES-1 inhibitor PF-03549184 had no effected on CSE-induced cell proliferation, it is plausible that the anti-proliferative effect of celecoxib could be mediated by its inhibition of TXA_2_ release in both cell types. To test this possibility, the effect of celecoxib on TXA_2_ release (measured as TXB_2_) was assessed. As shown in Fig. 7A, basal level of TXB_2_ was very high (about 6000 pg/mg protein) due to high levels of TXB_2_ in the medium with 20% FBS (for cell proliferation), and no further increase by CSE and no inhibition by celecoxib were observed in PASMCs. In contrast, TXB_2_ was also detected basally (about 700 pg/mg protein) in the culture medium for PAECs with 2% FBS and a significant increase was observed after CSE treatment, which was markedly reduced by celecoxib (Fig. 7B). The results suggest that celecoxib can inhibit CSE-induced TXA_2_ release from both PASMCs and PAECs, thereby contributing to its anti-proliferative effect and providing further evidence that TXA_2_ is a key mediator in CSE-induced pulmonary cell proliferation.

## Discussion

The main novel findings of the current study are that CSE induced imbalanced vasoactive gene expression and mediator release, particularly the reduced PGI_2_/TXA_2_ ratio in both PASMCs and PAECs mainly due to increased TXA_2_ production and that the restoration of PGI_2_ and TXA_2_ imbalance by beraprost sodium and celecoxib (via the inhibition of COX-2-derived TXA_2_ production) and the antagonism of TXA_2_ receptor by daltroban all inhibited CSE-induced cell proliferation in both PASMCs and PAECs. To the best of our knowledge, these findings are the first to suggest that PGI_2_ and TXA_2_ imbalance plays a key role in cigarette smoke-induced pulmonary vascular cell proliferation and consequently PGI_2_ analogues and inhibitors of TXA_2_ production and effect may prevent pulmonary vascular remodelling and ultimately have therapeutical potential in pulmonary hypertension associated with COPD.

COX-2 is regarded as a vasoactive gene, and its induction is important in mediating the production of its downstream prostanoid products that have potent anti-proliferative (e.g. PGE_2_ and PGI_2_) and proliferative (e.g. TXA_2_) properties [37]. The fact that CSE, in the present study, upregulated *PTGS2* mRNA and COX-2 protein expression in both PASMCs and PAECs strongly suggests that CSE regulates COX-2 expression via transcriptional regulation. Although in vitro studies have reported COX-2 upregulation by CSE in non-pulmonary artery cell types, such as human small airway epithelial cells [25], in endothelial cells, such as human umbilical vein endothelial cells [26] and human pulmonary microvascular endothelial cells [27], and in vivo studies have demonstrated COX-2 induction in the lung tissue of both patients with COPD and smokers without COPD compared with non-smokers without COPD [26], our study is the first to show that CSE induces COX-2 expression in PASMCs and PAECs, the two cell main types of pulmonary artery vasculature.

Since COX-2 is the rate-limiting enzyme in prostanoid synthesis, its upregulation is expected to lead to the increase in the production of its downstream prostanoid products, such as PGE_2_. The observation that CSE induced COX-2 expression as well as PGE_2_ production in PAECs supports this concept. This is further supported by our previous findings demonstrating that inflammatory mediators such as IL-1ß can induce the protein expression of COX-2 and its product PGE_2_ in human PASMCs [38]. Similar to our findings from PAECs, it has been reported that CSE can induce the expression of COX-2 and mPGES-1 and production of PGE_2_ in normal human lung fibroblasts [39]. Unexpectedly, there was a dissociation between increase of COX-2 expression and decrease of its downstream product PGE_2_ in response to CSE in PASMCs in our study. This finding, together with the increased PGE_2_ levels by CSE in PAECs, suggests that CSE may regulate differently in PASMCs and PAECs the expression of mPGES-1, the enzyme that mediates PGE_2_ conversion from the COX-2 product PGH_2_. The fact that mPGES-1 expression was reduced in PASMCs, but unchanged in PAECs, strongly supports the concept that mPGES-1 downregulation plays a crucial role in mediating the reduced PGE_2_ production in PASMCs by CSE, despite the increased COX-2 expression. Although PGE_2_ is poorly characterized as a key factor of pulmonary artery cell dysfunction in all forms of pulmonary hypertension, there is evidence to suggest a potential role of dysregulated PGE_2_ in the development of group 1 pulmonary hypertension. For instance, it has been reported that circulating PGE_2_ levels are reduced in patients with group 1 pulmonary hypertension [22] and that highly selective EP2 receptor agonist butaprost is able to inhibit proliferation of human PASMCs derived from patients with group 1 pulmonary hypertension [23]. However, since CSE reduced PGE_2_ production in PASMCs but increased PGE_2_ production in PAECs, similar inhibition on CSE-induced cell proliferation in both cell types by celecoxib, which inhibits production of all prostanoids downstream of COX-2, including PGE_2_, suggests that PGE_2_ is not critically involved in CSE-induced PASMC and PAEC proliferation. The fact that mPGES-1 inhibitor PF-03549184 did not have any effect on CSE-induced PASMC and PAEC proliferation (unpublished observation) provides further evidence that other prostanoids, such as PGI_2_ and TXA_2_, may play a more important role than PGE_2_ in CSE-induced cell proliferation. However, the exact role of CSE-mediated reduced PGE_2_ in PASMCs in cigarette smoke-induced pulmonary vascular remodelling requires further investigation.

PGI_2_ plays a key role in maintaining local vascular tone. It has been reported that urinary metabolites of PGI_2_ are decreased in group 1 pulmonary hypertension [24]. A reduction of PGIS expression and PGI_2_ production has also been demonstrated in lung tissue of patients with COPD [27]. Importantly, in vitro studies showed that the use of PGI_2_ analogue can prevent CSE-induced cell apoptosis in human pulmonary microvascular endothelial cells [27] and umbilical vein endothelial cells [28], indicating a potential role of decreased PGI_2_ levels in cigarette smoke-induced pulmonary endothelial cell dysfunction and apoptosis in patients with COPD. Our study is the first to explore the effect of CSE on PGIS-derived PGI_2_ and contribution of PAECs, particularly PASMCs in this process. We showed that *PTGIS* mRNA expression, PGIS protein expression and PGI_2_ production were all reduced by CSE in PASMCs. This finding, together with the inhibition of CSE-induced PASMC proliferation by the PGI_2_ analogue beraprost sodium, strongly suggests that reduced PGIS/PGI_2_ plays a key part in CSE-induced imbalanced vasoactive prostanoid production in PASMCs and may ultimately contribute to pulmonary vascular remodelling in COPD. *PTGIS* mRNA expression was also reduced by CSE in PAECs. Surprisingly, PGIS protein expression was undetectable by Western blotting in both untreated and CSE-treated PAECs, suggesting that PAECs constitutively express less PGIS than PASMCs. This is supported by the fact that PAECs constitutively produced less PGI_2_ than PASMCs (792.0 ± 94.7 pg/mg protein compared with 2987.2 ± 403.5 pg/mg protein). It is also reasonable to speculate that CSE had no effect on PGIS protein expression since PGI_2_ release was unchanged. These findings in PAECs are in disagreement with the previous in vitro study showing reduced PGI_2_ release in response to CSE (24 h, up to 10%) in human umbilical vein endothelial cells [28]. The discrepancies may be due to the differences in cell type and cigarette brand (Marlboro) used in the other study. In addition, our findings in PAECs also differ from those in PASMCs demonstrating reduced PGIS expression and PGI_2_ production by CSE. This may be explained by the differences in cell types and in CSE treatment time (24 h for PAECs and 72 h for PASMCs). Although exogenous PGI_2_ exerted anti-proliferative effect on CSE-induced PAEC proliferation, the unchanged PGI_2_ production following CSE treatment in PAECs suggests that PGI_2_ may not play a key role in CSE-induced imbalanced vasoactive prostanoid production and cell proliferation in PAECs.

It is worth noting that although it is commonly considered that PAECs are the main source of PGI_2_, the facts that PASMCs produce higher basal levels of PGI_2_ than PAECs and that CSE-induced imbalanced vasoactive prostanoid mediator release is more prominent in PASMCs than in PAECs suggest that PASMC dysfunction may be more important than the traditionally recognized PAEC dysfunction in contributing to pulmonary artery remodelling and pulmonary hypertension in COPD. Although reports have shown other cell types (e.g. adventitial fibroblasts) in the pulmonary vasculature can also contribute to vascular remodelling [13], their role in COPD-associated pulmonary hypertension in response to cigarette smoke has not been explored.

Our findings that CSE induced COX-2 expression but reduced both PGI_2_ and PGE_2_ production in PASMCs as a result of PGIS and mPGES-1 downregulation, respectively, strongly suggest that CSE can selectively reduce vasodilatory and anti-proliferative gene expression and mediator release in prostanoid biosynthesis and that the downregulated PGIS and mPGES-1 expression may divert the COX-2 product PGH_2_ away from being converted to PGI_2_ and PGE_2_ towards the systhesis of the vasoconstrictive and proliferative mediator TXA_2_ via TXAS. To our knowledge, this study is the first to assess CSE effect on TXAS-derived TXA_2_ in human PASMCs and PAECs. Although TXAS protein expression was undetectable in both untreated and CSE-treated PASMCs and PAECs despite increased *TBXAS1* mRNA expression, it is highly likely that TXAS protein was below the detection of Western blotting under both conditions. This is supported by our findings that hypoxia (1% O_2_) induced TXAS expression using the same human PASMCs (unpublished observation) and that TXAS was detected at basal levels and was further induced in CSE-treated normal human bronchial epithelial cells (positive control). Since *TBXAS1* mRNA and TXAS product TXA_2_ were detected in unstimulated PASMCs and PAECs and were increased by CSE in both cell types, it is likely that TXAS protein expression was increased in response to CSE, although the possibility that TXAS protein expression was unchanged or decreased could not be excluded.

Despite the undetected TXAS protein expression, the increase of TXA_2_ by CSE suggests it is the result of the conversion by the existing TXAS of the increased PGH_2_, due to the combined effect of CSE on COX-2 upregulation (both cell types), PGIS and mPGES-1 downregulation (PASMCs) and unchanged PGIS expression (PAECs). It is also possible that CSE may increase TXAS activity (e.g. by post-translational modifications) rather than its expression, although there is no direct evidence. The increase of vasoconstrictive and proliferative mediator TXA_2_ by CSE in both cell types suggest that TXA_2_ may act as a key mediator of pulmonary artery cell dysfunction in COPD. This is supported by the ability of the TXA_2_ receptor antagonist daltroban to inhibit CSE-induced PASMC and PAEC proliferation, which also suggests that blocking TXA_2_ effect may reduce pulmonary vascular remodelling induced by cigarette smoking. The fact that selective inhibition of COX-2 by celecoxib significantly reduced CSE-induced TXA_2_ production in PAECs, but not in PASMCs due to the interfernce of the assay by existing high levels of TXB_2_ in FBS (20%) in the medium used for cell proliferation, strongly suggests that the anti-proliferative effect of celecoxib is the result of the inhibition of COX-2-derived TXA_2_ production and provides further evidence that CSE-induced COX-2 expression may have a detrimental effect on pulmonary vascular remodelling by mediating the induction of imbalanced prostanoid production, particularly increased TXA_2_ levels.

Together with TXA_2_, PGI_2_ plays an important role in maintaining homeostatic balance in pulmonary circulation. In the present study, we assessed CSE effect on PGI_2_/TXA_2_ ratio and found, for the first time, that CSE reduced the vasodilatory and anti-proliferative mediator PGI_2_ and increased the vasoconstrictive and proliferative mediator TXA_2_ in PASMCs, resulting in a reduced PGI_2_/TXA_2_ ratio. Although CSE had no effect on PGI_2_ production in PAECs, CSE also reduced the PGI_2_/TXA_2_ ratio in PAECs due to increased TXA_2_ production. These observations suggest that CSE can cause PASMC and PAEC dysfunction by inducing an imbalance between PGI_2_ and TXA_2_, thereby contributing to cigarette smoke-induced vascular remodelling in COPD and that agents that target the imbalance by either compensating for PGI_2_ reduction or blocking TXA_2_ effects may have therapeutic potential in COPD-associated pulmonary hypertension.

## Conclusions

In conclusion, our findings that CSE induced an imbalanced prostanoid release characterised by the reduced PGI_2_/TXA_2_ ratio, likely as a result of an imbalanced expression of PGIS and TXAS, and that agents targeting the imbalance inhibited CSE-induced pulmonary artery cell proliferation point to TXA_2_ as a novel mediator of pulmonary artery cell dysfunction and proliferation in pulmonary hypertension in COPD and strongly suggest that targeting the imbalance may have therapeutic potentials for this fatal disease and that smoking cessation may prevent further vascular remodelling.

## Data Availability

All data generated and analyzed during this study are available from the corresponding author upon reasonable request.

## Abbreviations

CSE: Cigarette smoke extract
COPD: Chronic obstructive pulmonary disease
PASMCs: Pulmonary artery smooth muscle cells
PAECs: Pulmonary artery endothelial cells
ECGM: Endothelial cell growth medium
COX-2: Cyclooxygenase-2
PG: Prostaglandin
TX: Thromboxane
PGIS: PGI synthase
TXAS: TXA synthase
mPGES-1: Microsomal PGE synthase-1
AA: Arachidonic acid
*PTGS2*: Prostaglandin-endoperoxide synthase 2 gene
*PTGIS*: PGI_2_ synthase gene
*TBXAS1*: TXA synthase 1 gene
*β2M*: β_2_-Microglobulin gene
WST-1: Water soluble tetrazolium salt 1
